# Characterisation of temperature-dependent phase transitions in 2,2-trimethylenedioxy-4,4,6,6-tetrachlorocyclotriphosphazene, N_3_P_3_Cl_4_[O(CH_2_)_3_O]

**DOI:** 10.1186/1752-153X-1-20

**Published:** 2007-07-18

**Authors:** Simon J Coles, David B Davies, Michael B Hursthouse, Susanne L Huth, Adem Kılıç, Mark E Light, Marianne Odlyha, John S Rutherford, Robert A Shaw, Aylin Uslu

**Affiliations:** 1 School of Chemistry, University of Southampton, Highfield, Southampton. SO17 1BJ, UK; 2 School of Biological and Chemical Sciences, Birkbeck College (University of London), Malet Street, London WC1E 7HX, UK; 3 Department of Chemistry, Gebze Institute of Technology, Gebze, Turkey; 4 1 Barliath, Minard, Argyll PA32 8YQ, UK

## Abstract

**Background:**

The crystal structure of 2,2-trimethylenedioxy-4,4,6,6-tetrachlorocyclo triphosphazene has been determined at 120, 274 and 293 K. The result at 293 K confirms the room temperature *Cmc2*_1 _structure, but at the lower temperatures the space group is *Pna2*_1_. Nevertheless the basic structure remains the same, with only small displacements of the atoms, amounting to an average of 25 pm between 120 and 293 K.

**Results:**

X-ray diffraction and DSC results indicate that the phase transition takes place in two steps between 274 – 293 K and provides an understanding of previous NQR results. In the intermediate temperature range the molecules are displaced from their room temperature positions in such a way as to give an average structure with *Cmc2*_1 _symmetry.

**Conclusion:**

The overall phase transition is consistent with the occurrence of a soft lattice mode at room temperature in which a large displacement of the molecule in the *x*-direction is coupled with a flexing motion about an axis defined by the nitrogen atoms in the N1 and N3 positions.

## 1. Background

**Scheme 1:**
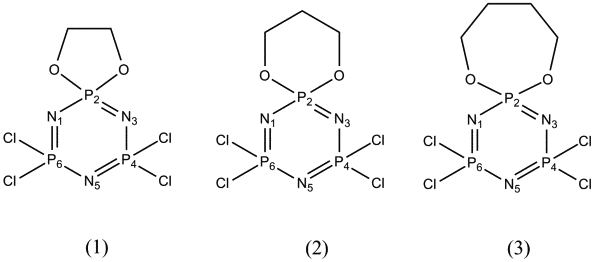


The room temperature crystal structures of the series of mono-spiro compounds containing 5-, 6- and 7-membered phosphate rings, N_3_P_3_Cl_4 _[O(CH_2_)_2_O], (**1**) N_3_P_3_Cl_4 _[O(CH_2_)_3_O] (**2**) and N_3_P_3_Cl_4 _[O(CH_2_)_4_O] (**3**) respectively, were reported by Contractor et al [1]. The ^35^Cl Nuclear Quadrupole Magnetic Resonance (NQR) spectra of these compounds were subsequently measured over a temperature range from approximately 100 K to 300 K [2]. Compound **1 **gave rise to four different ^35^Cl NQR signals over the whole range of temperature with no discontinuities being apparent. The crystal structure of **1 **[1] revealed a twist-boat conformation of the cyclophosphazene ring with four structurally-distinguishable Cl atoms mirrored in the ^35^Cl NQR spectrum. Compound **3 **showed only two ^35^Cl NQR signals over the whole temperature range with a slight cusp near 120 K. Its crystal structure at room temperature revealed that this compound possessed a diad axis of symmetry along the P-N axis formed by the atoms in the 1 and 4 positions, with only two crystallographic distinct Cl atoms, in keeping with the NQR spectroscopic observations. Earlier NQR measurements on other cyclotriphosphazene derivatives had shown the frequent occurrence of cusps near 150 K indicating minor changes in the solid state at this temperature, although the nature of these cusps was not explained [3]. By contrast, variable temperature NQR measurements on compound **2 **showed four different ^35^Cl signals at lower temperatures from 90 K up to approximately 260 K, where the signals from these nuclei disappeared and then reappeared as two signals at approximately 280 K and higher temperatures. The room temperature crystal structure of compound **2 **showed the molecule to have a mirror plane, which passes through the plane of the six-membered N_3_P_3 _ring, thus giving only two unique Cl atom environments. Obviously the low temperature structure had a lower symmetry, thus giving rise to four ^35^Cl NQR signals from four crystallographically-different Cl atoms. At that time technical difficulties prevented both a low temperature and variable temperature study by X-ray crystallography. In the present study, the phase transition observed by NQR for compound **2**, has been investigated by X-ray crystallographic and thermal methods to provide greater understanding of the process (see scheme 1).

In order to maintain consistency with previous work on compounds **1 **– **3 **[1], we have used the original 2,4,6 numbering of the phosphorus atoms for the cyclophosphazene ring rather than change to the 1,3,5 numbering using IUPAC convention.

## 2. Experimental

### 2.1. Synthesis and crystallisation

Compound **2 **was prepared as in the literature [4], characterised by ^1^H and ^31^P NMR [5] and was crystallised from dichloromethane. The melting temperature of compound **2 **in the literature [1] is given as 429–430 K but is measured as 427 K by Differential Scanning Calorimetry (DSC) in this work.

### 2.2 Differential Scanning Calorimetry

Thermal measurements were made using a Shimadzu Differential Scanning Calorimeter (DSC60). Samples were examined under a polarising Olympus microscope at 100× and good quality crystals were selected for the measurements. The selected crystals were then placed in custom made Al micro-crucibles and weighed on a Sartorius electronic microbalance. Since attention was paid to crystal quality the sample size used was small (0.326 mg). A second sample (1.982 mg) was also tested. Cooling to 323 K was performed using liquid nitrogen and samples were heated to room temperature at 5 K/min. A total number of 5 cycles were performed of cooling and heating. After the 5^th ^cycle samples were melted by heating at 5 K/min to 438 K and then cooled to liquid nitrogen temperatures and then re-heated to room temperature. Nitrogen was used as purge gas with a flow rate of 60 cm^3^/min. The reference crucible was an empty Al micro-crucible.

### 2.3. Structure determinations

Intensity data were recorded on a Nonius KappaCCD diffractometer situated at the window of Bruker Nonius FR591 rotating anode generator equipped with a molybdenum target (λ Mo-kα = 0.71073 Å) and driven by COLLECT [6] and DENZO [7] software. Structures were determined using the direct methods procedure in SHELXS-97 and refined by full-matrix least squares on F^2 ^using SHELXL-97 [8]. Data were corrected for absorption effects by means of comparison of equivalent reflections using the program SADABS [9]. Non-hydrogen atoms were refined anisotropically, whilst hydrogen atoms were fixed in idealised positions (0.99 Å) with their displacement parameters riding on the values of their parent atoms (1.2 *U*_*eq*_). The temperature of the sample was controlled using an Oxford Cryosystems 700 series Cryostream Plus cryostat [10]. Pertinent data collection and refinement parameters are openly available for download for the 120 K [11] (for CIF data see Additional File 1), 274 K [12](for CIF data see Additional File 2) and 293 K [13] structures (for CIF data see Additional File 3). CIF data has also been deposited with the Cambridge Crystallographic Data Centre (deposition numbers CCDC641683 – CCDC641685).

**Figure 1:**
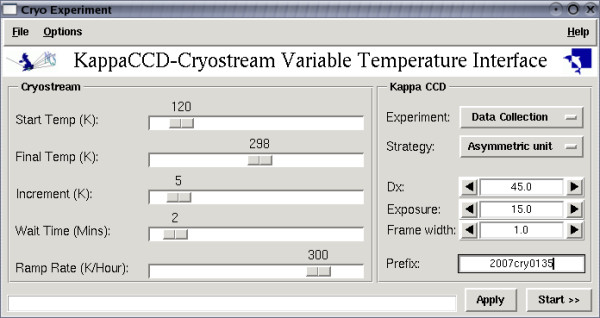
The interface for the CRYOGUI software.

To fully characterise the solid state thermal behaviour of compound **2 **by single crystal X-ray diffraction, unit cell data were collected at 5 K increments, at a rate of 2 K/min in the temperature range 170 K to 400 K (some 30 K below the melting point) and are submitted as Additional file 4. The system was subjected to an automated regime whereby the temperature is systematically raised and data collected as implemented in the new software, CRYOGUI, described herein (see figure 1). The data collection procedure is carried out in a stepped isotherm fashion i.e. the temperature is ramped to a target, allowed to equilibrate for 1 minute and then held for approximately 2 minutes whilst the unit cell data is acquired and then the next ramping and data collection cycle performed.

Although a number of diffractometer manufacturers provide rudimentary control of low temperature devices via their data collection software, it is generally not flexible enough to carry out complex variable temperature experiments in an automated fashion. To address the problems posed by the chemistry under investigation in this study, it was necessary to write software that could automate the collection of multiple data sets under various temperature-change regimes. The application is written in Python and utilizes the Nonius Python libraries of the COLLECT [6] software, along with the associated *programmer's manual*. The libraries include interfaces to both the KappaCCD and the Oxford Cryosystems cryostream enabling a synergistic approach to the experiment. The software allows the following variables to be controlled; start temperature, final temperature, increment (determines the number of steps), ramp rate and dwell time (to allow a crystal to equilibrate at its new temperature). The type of diffraction experiment can be set as either full data collection or unit cell determination, and the usual choices of experimental parameters are available. In order to avoid time consuming processing of multiple data sets, data reduction is carried out automatically and therefore, once the experiment has been designed, it can be left to run with no further human input.

## 3. Results and discussion

### Phase transition in compound 2

**Figure 2:**
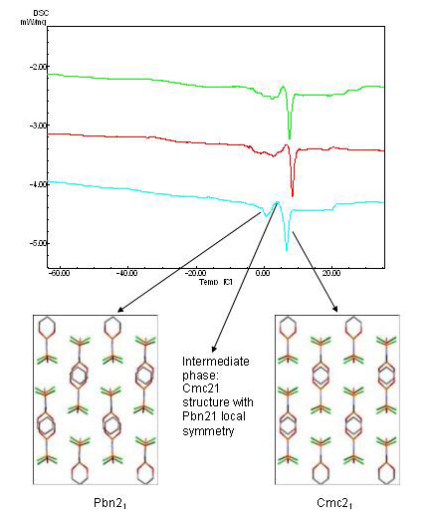
Differential Scanning Calorimetry plot, showing three cycles (green = cycle 1, red = cycle 2, blue = cycle 3) in the region of the phase transition and the correlation between thermal behaviour and structure (viewed down the c axis).

The single crystal (0.326 mg) was first cooled to 143 K in the calorimeter and the DSC measured to 323 K revealing the presence of an endothermic event over a broad temperature range (263 – 283 K), which coincides with the temperature region where a discontinuity had been previously observed in ^35^Cl NQR measurements [2]. Successive cooling and re-heating showed the gradual emergence of two endothermic peaks (ca. 273 and 280 K) in this temperature range, becoming identifiable by the third re-heat and then the peaks gradually sharpened and became better defined on the further two cycles of cooling-heating. Three of the DSC runs are shown in Figure 2, where the green trace corresponds to the 3^rd ^re-heat, the red trace to the 4^th ^re-heat, and the blue trace to the 5^th ^re-heat; for the latter the 1^st ^endothermic peak (ca. 273 K) occurs over a relatively narrow temperature range and is followed immediately by a crystallisation peak (exothermic, ca. 277 K) and then by the much narrower second endothermic peak (ca. 280 K). The thermal behaviour of the 5^th ^re-heat indicates the presence of an intermediate phase and we assume that the two endothermic peaks define the temperature range of the intermediate structure. The melting of this thermally-cycled sample is a sharp single peak (melting temperature 427 K) as shown in Figure S1 (see Additional File 5), and which indicates a high level of purity of the sample. Further cooling of the melted material to 143 K, crystallisation and then re-heating to room temperature revealed once more the presence of the two endothermic transitions *ca*. 273 and 280 K. DSC measurements on a larger sample (1.982 mg) showed the presence of two endothermic peaks even on the first heating from 143 K to 313 K and further cycling showed an increase in intensity of the first endothermic peak until both endothermic peaks were about equivalent (these traces are provided as Figure S2 in Additional File 5, and, although little annealing is required, the results show that the bulk material behaves in a similar fashion to the single crystal). The DSC therefore shows that the phase change behaviour is considerably more complex than a single step transition and a variable temperature single crystal diffraction experiment was designed in order to gain further insight into its mechanism. Crystal structures were determined at temperatures of 120 K, 274 K and 293 K and unit cells were determined in an automated variable temperature experiment at 5 K intervals between 170 K and 400 K.

Although there is a very simple relationship between the low and room temperature structures of this compound, with one structure realisable from the other with only very small displacements of the atoms, the phase transition does not appear to follow the simple second-order model of Landau but is more complex, taking place in at least two stages over a temperature range of about fifteen degrees. The phenomenon of a two-stage transition occurring for such a simple symmetry relationship has been recognised previously [14]. In such cases it is believed that there is an intermediate phase, in which there is a long range order corresponding to the high-temperature structure, but the local symmetry conforms to the low-temperature phase, with fluctuations occurring.

In order to compare the low temperature structure to the room temperature structure in *Cmc2*_1_, it must first be transformed to a non-standard setting of space group *Pna2*_1_, namely *Pbn2*_1_, by interchanging the *a*- and *b*-axes. Then, using International Tables [15] we find these two space groups have a simple maximal index-2 group-subgroup relationship, which is class-equivalent rather than translation-equivalent, and so the primitive unit cell must double in volume in going from room temperature to low temperature. (However, since the room temperature space group is *C*-centred, the conventional cell remains the same.) This symmetry relationship is borne out by the actual structures, since the low-temperature structure is realisable from the other with only small displacements of the atoms, during which the asymmetric unit doubles and the molecular mirror plane disappears. In terms of the Wyckoff positions, atoms occupying the special positions (4a) in *Cmc2*_1 _with fixed *x*-coordinate occupy general positions (4a) in *Pbn2*_1 _with no such restriction, while atoms in the general positions (8b) of *Cmc2*_1 _split into two separate sets of general positions (4a) in *Pbn2*_1_.

If we apply the phenomenological theory of such transitions [14], we can recognise two order parameters which might contribute to the Gibbs energy of the system and lead to this behaviour. Firstly we note that the displacements leading to the low-temperature structure arise in an ordered manner, such that molecules separated by the vector (1/2, 1/2, 0), related by a lattice translation at room temperature, are displaced in opposite directions in the low temperature structure, where they are related only by a glide operation. Such an effect can arise in a "soft mode" transition, so-called [16] since it can be explained in terms of a temperature dependence of the frequency of a normal mode of crystal vibration. At some temperature there ceases to be a restoring force associated with that particular set of atomic displacements, since the potential well changes from having a symmetric minimum corresponding to the atomic positions at room temperature to having a symmetric double minimum. When the resulting instability becomes sufficiently pronounced, the crystal transforms co-operatively to one of the two possible lower symmetry states with equivalent structures.

Since the group-subgroup relationship is not to a translation-equivalent subgroup, we can locate where the propagation vector of this mode lies in reciprocal space away from the Brillioun zone origin, and so correlate it with the actual atomic displacements. Specifically, since the additional Bragg reflections of the low-temperature phase occur for (*h *+ *k*) odd, the displacements of pairs of atoms (1/2, 1/2, 0) apart in the unit cell must be 180 degrees out-of-phase in the normal mode concerned. Given the loss of the mirror plane present in the high-temperature structure, we expect them to lie perpendicular to this plane, i.e. along the *a*-axis.

An attempt was made to describe the displacements in the low temperature phase in terms of the symmetry mode analysis of Aroyo and Perez-Mato [17]. In order to apply this analysis to a molecule, we could consider it as a rigid body, in which case it will give rise to just six lattice modes. However this makes the assumption that any internal deformations are so stiff as to have insignificant amplitude as compared to the lattice modes. This is not necessarily the same as saying that the free molecule can adopt only one or several conformations; for, as we shall see in this case, a moiety normally considered rigid may show significant deformation, or one capable of adopting several conformations remain unaffected through the transition.

Now three of these lattice modes involve displacements of the centre of mass, which correlate with the translations of the free molecule. These can be treated in an identical way as for an individual atom by the Aroyo-Perez-Mato method. However the other three are librational modes, which correspond to rotations of the free molecule. Taking as an example a libration *L*_*x *_about the *x*-axis, we may treat these by applying the method to two sets of Wyckoff positions, the one which contains the point (*x*, *y *+ *v*, *z*) and the one containing (*x*, *y*, *z *+ *w*), where *v *and *w *are small displacements. In order to carry out this analysis, since the three structure determinations chosen for comparison had each been refined independently in the standard setting of the appropriate space group, the cell axes and coordinates of the low temperature structures were transformed to bring each of the reference molecules into correspondence with each other and with the reference molecule at room temperature, fixing as identical the *z*-coordinates of the centre of mass in all three cases.

The centre of mass was found to be displaced along *x *by 25 pm at 120 K and 17 pm at 274 K. Since the displacements of the atoms from their room temperature positions at 274 K proved to be uniformly two-thirds of those at 120 K, the subsequent analysis concentrated on comparing the structures at 120 K and 293 K. No satisfactory fit could be found of the low temperature molecule to the room temperature molecule by simple rotational displacements about the centre of mass. This difficulty was traced to an internal deformation of the molecule, when it became clear from the observed torsion angles that the molecule at 120 K was folded along the N1 – N3 axis. Using this model to fit the internal non-bonded distances indicated a folding angle of 9.1°, which was apportioned in transforming the coordinates in the ratio 0.35 : 0.65, the inverse ratio of the relevant moments of inertia about N1 – N3. After readjusting to the centre of mass, the positions of the non-hydrogen atoms in the two structures could be brought into good agreement (standard error 5 pm) by very small rotational adjustments.

**Figure 3:**
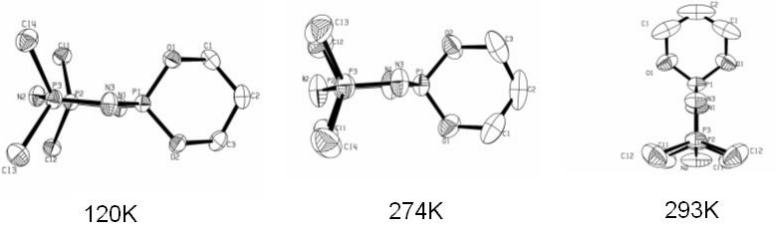
Thermal ellipsoid plots (30% probability) of a molecule viewed along the *c*-axis at (from left) 120, 274 and 293 K.

The displacements observed in the low-temperature structure are completely consistent with this soft mode model and the thermal ellipsoids in the various determinations, as modelled by ORTEP [18] and shown in Figure 3, reveal the enhanced thermal motion directly. At room temperature they are extremely elongated along *a*. At 120 K they are much closer to spherical and quite normal in form, but at 274 K, as the transition is approached from the low temperature side, they also show greatly enhanced motion along the *a*-axis, consistent with there being a very shallow but asymmetric minimum in the potential function at that temperature.

The other phenomenon that should be considered is macroscopic strain, *i.e*. there may be a ferroelastic aspect to the phase transition. Elongation or compression of the crystal in the appropriate direction should alter the form of the potential function for the normal mode in question. Since the displacements associated with the soft mode lie along *a*, we would predict the direction of maximum thermal expansion to lie in that direction, and as well as any actual isothermal shear. The lattice parameters were compared [19] over the range 200 to 320 K. The thermal expansion remains fairly uniform in the range 200 to 290 K with principal components of the expansivity tensor α_a _= 99(3), α_b _= 45(3) and α_c _= 44(3) × 10^-6^ K^-1^, with α_a _being by far the largest as would be expected. However there also appears to be a small isothermal shear, affecting mainly the *a*-axis, at about 295 K (full data and plots of unit cell length as a function of temperature are provided in Figure S3, see Additional File 5).

## 4. Conclusion

The NQR [2] clearly shows the relative symmetries of the room and low temperature phases in the number of lines observed, and the way the corresponding lines appear to converge supports a model where there are no abrupt changes across the transition. However the lines broaden to the point of disappearance over a temperature range of about 20 K, from a little below the lower transition to near the upper transition temperature. This suggests that the fluctuations present in this temperature range must be such as to produce a very large range of individual chlorine atom environments.

The DSC measurements indicate that the transition occurs as a two stage process with an intermediate crystallisation event. The transition is observed to be reproducible and become sharper as the sample is annealed on cycling. A sharp melting point is observed and the behaviour of the bulk material is shown to mimic that of the small amount of pure material.

**Figure 4:**
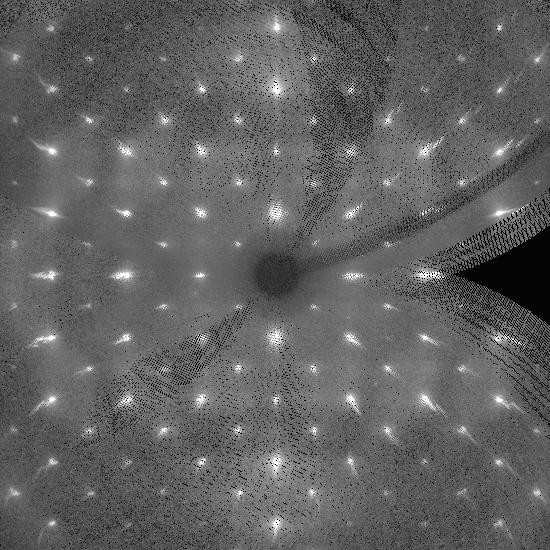
A simulated precession photograph of the (*hk*0) zone of the intermediate structure.

As for X-ray diffraction, we observe in the transitional region a diffraction pattern with the presence of strong disorder diffuse scattering. For example the *hk*0 projection contains additional weak and diffuse spots near the positions of the (*h *+ *k*) odd reflections which appear at low-temperature, superimposed on a sharp high-temperature pattern (Figure 4). This effect is so marked that initial attempts to determine the space group automatically in this region were frustrated by ambiguities.

The various observations may be interpreted in the following way in terms of cooling the crystal. Above room temperature the molecule lies in a crystallographic mirror plane in space group *Cmc2*_1_, but there are large vibrational displacements in the *x*-direction, which couple to a flexing motion along the long molecular axis. At approximately 290 K there is a weak first order isosymmetric transition to an intermediate state in which the molecules occupy two symmetry related positions in close proximity to each other, *i.e*. the Wyckoff positions of *Cmc2*_1 _break down to those of *Pbn2*_1_, but these are occupied in a disordered fashion so as to maintain on average *Cmc2*_1 _symmetry.

As the temperature is lowered further through this intermediate region, the two molecular sites move steadily apart, and their correlation in terms of the displacement directions of neighbouring molecules becomes progressively longer in range, until about 274 K the system undergoes a second weak first order transition, where the crystal assumes the ordered low temperature structure in *Pbn2*_1_.

## Supplementary Material

Additional file 1 120 k CIF data. Click here for file

Additional file 2 274 k CIF data. Click here for file

Additional file 3 293 k CIF data. Click here for file

Additional file 4 Supplementary data. Click here for file

Additional file 5 Supplementary information. Click here for file
