# Supplementary material for: Characterisation of temperature-dependent phase transitions in 2,2-trimethylenedioxy-4,4,6,6-tetrachlorocyclotriphosphazene, N3P3Cl4[O(CH2)3O]
Source: Chem Cent J. 2007 Jul 18;1:20. doi: 10.1186/1752-153X-1-20 (PMC1989690; doi:10.1186/1752-153X-1-20)
Supplement: Additional file 5 — Supplementary information. [file 1752-153X-1-20-S5.doc]

Supplementary Information

S1) DSC of compound 2, showing pure phase melting point.

S2) DSC cycles of bulk material

S3) Unit cell data over 200 - 320K temperature range.
